# Continuous-Flow Synthesis of Nitro-*o*-xylenes: Process Optimization, Impurity Study and Extension to Analogues

**DOI:** 10.3390/molecules27165139

**Published:** 2022-08-12

**Authors:** Qiao Song, Xiangui Lei, Sheng Yang, Sheng Wang, Jianhui Wang, Jiujun Chen, Yong Xiang, Qingwu Huang, Zhouyu Wang

**Affiliations:** 1 Department of Chemistry, Xihua University, Chengdu 610039, China; 2 Asymmetric Synthesis and Chiral Technology Key Laboratory of Sichuan Province, Yibin 644000, China; 3 Yinguang Group Sichuan North Hongguang Special Chemical Co., Ltd., Yibin 644000, China

**Keywords:** continuous-flow, nitration, *o*-xylene, process optimization, impurity study

## Abstract

An efficient continuous-flow nitration process of *o*-xylene at pilot scale was demonstrated. The effects of parameters such as temperature, ratio of H_2_SO_4_ to HNO_3_, H_2_SO_4_ concentration, flow rate, and residence time on the reaction were studied. Under the optimal conditions, the yield of products reached 94.1%, with a product throughput of 800 g/h. The main impurities of this continuous-flow nitration process were also studied in detail. Compared with batch process, phenolic impurity decreased from 2% to 0.1%, which enabled the omission of the alkaline solution washing step and thus reduced the wastewater emission. The method was also successfully applied to the nitrification of *p*-xylene, toluene, and chlorobenzene with good yields.

## 1. Introduction

**Figure 1:**
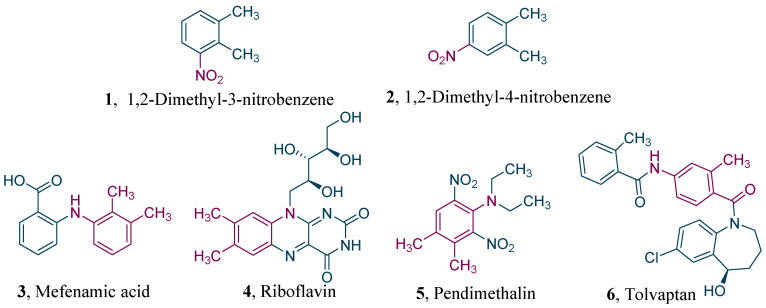
Structures of nitro-*o*-xylenes and related downstream products.

**Figure 2:**
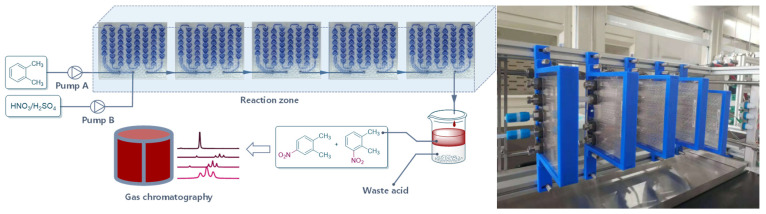
Continuous-flow reactor setup for condition optimization.

**Figure 3:**
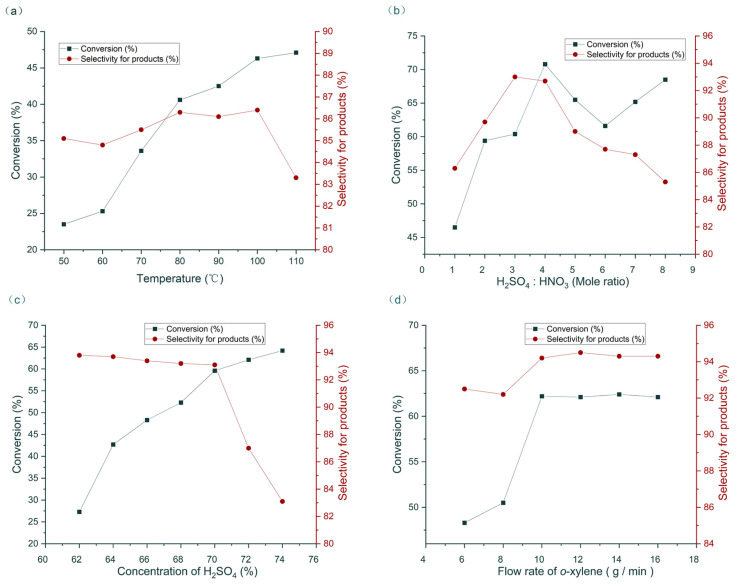
Optimization of nitration in continuous flow. (**a**) The influence of temperature; (**b**) the influence of mole ratio of H_2_SO_4_ and HNO_3_; (**c**) the influence of H_2_SO_4_ concentration; (**d**) the influence of flow rate.

**Figure 4:**
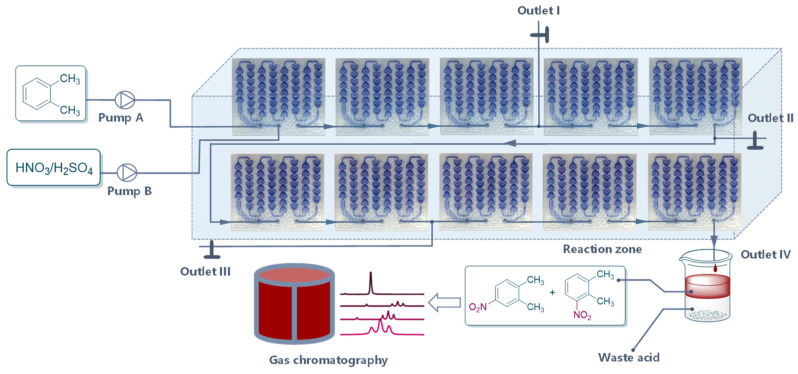
Continuous-flow reactor setup for the inspection of residence time.

**Figure 5:**
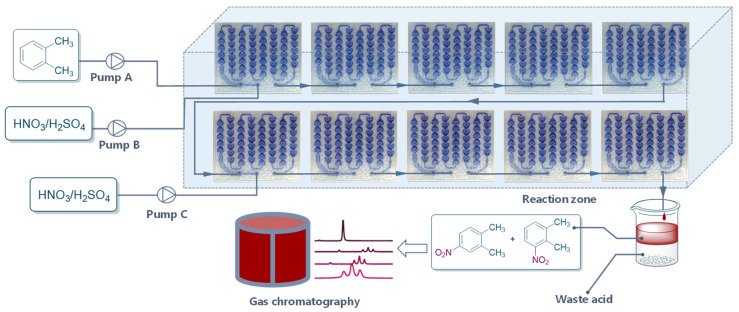
Continuous-flow reactor setup for the optimal process.

**Figure 6:**
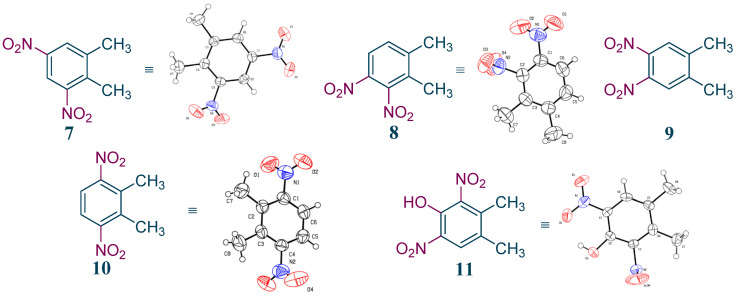
The structures of the main impurities in *o*-xylene nitration process.

Nitro-*o*-xylenes are important ingredients and intermediates applied broadly in pharmaceuticals, agricultural chemicals, dyes, and many other areas. For instance, 1,2-dimethyl-3-nitrobenzene (Figure 1, **1**) is the raw material of mefenamic acid [1] (Figure 1, **3**), an effective nonsteroidal anti-inflammatory drug. Meanwhile, 1,2-dimethyl-4-nitrobenzene (Figure 1, **2**) is extensively used to produce medicines such as riboflavin [2] (Figure 1, **4**), the herbicide pendimethalin [3] (Figure 1, **5**), and the cardiovascular drug tolvaptan [4] (Figure 1, **6**).

Nitro-*o*-xylenes are primarily synthesized by the nitration of *o*-xylene. Among the versatile methods [5], nitration with mixed acid [6,7,8,9] is the most commonly used one in the industry due to its economy and maturity. Although scientists have reported several greener strategies, such as using NO_2_ [10,11,12] and N_2_O_5_ [13] as nitrating agents or using acidic solid catalysts [14,15] to replace liquid acids, these methods suffer from problems, such as using expensive catalytic systems, difficulty of scaling up, and ensuring the process safety. Therefore, these new methods remain inferior to mixed-acid nitration as the predominant technique used in industries.

Obviously, nitration with mixed acid has its limitations. First, nitration using this method produces a large amount of waste acid, which causes environmental stress. This problem can be solved to some extent by recycling the waste acid, although extra expenses are incurred. Second, nitration reactions are highly exothermic [16]. The production of nitrocompounds using a traditional tank reactor usually generates large amounts of liquid holdup and has poor heat-transfer efficiency. Nitrocompounds are also usually explosive, and their raw materials are inflammable. These characteristics make nitration reaction extremely hazardous. The safe generation of nitration products in industries is urgently needed, and continuous-flow reactors may serve as powerful tools to achieve this goal.

Over the past decades, continuous-flow reactors have been greatly developed and extensively applied in organic syntheses [17,18,19,20,21]. The characteristic properties of these reactors are their exceptionally fast heat and mass transfer. Thus, the accumulation of heat, formation of hot spots, and dangers of thermal runaways can be prevented. Moreover, the small reactor volumes result in the significantly improved overall safety of the process, even under harsh reaction conditions [22]. Therefore, continuous-flow reactors are suitable for nitration.

The first report about the application of continuous-flow technology to nitrition reactions date back to 1956 [23]. Since then, continuous flow nitrition reactions have been developed rapidly [24,25,26,27,28,29,30,31]. There are also some elegant studies in continuous-flow nitrification of xylenes. In 2015, Kulkarni’s group studied an efficient continuous-flow nitration process of *o*-xylene using a tubular reactor [32]. In this process, 99% conversion of *o*-xylene and 7.2% dinitro impurities were observed using only fuming nitric acid (FNA) as the nitrating agent. However, up to six times as much FNA as *o*-xylene was used, which was a tremendous waste, and the best yield of nitro-*o*-xylenes was calculated to be 91.8%, which still has room for improvement. In 2021, Watts’s group disclosed a biphasic continuous-flow nitration procedure to generate various nitrobenzenes in a sonicated PTFE tube reactor and Uniqsis chip reactor [33]. Although the nitration in the sonicated PTFE tube reactor gave excellent yields, this method was not easy to scale up. Nitration in a Uniqsis chip reactor is much easier to scale up to decagram scale per hour, but the yield of nitro-*o*-xylenes is only 88%, which is unsatisfactory. Very recently, Li’s group designed an efficient two-step mononitration method of *m*-xylene in a microreactor [34]. Under the optimal experimental conditions, the yield of mononitro *m*-xylenes reached 99% and a throughput of 1 kg/h. However, this method requires the use of CH_2_Cl_2_ as a solvent, which is not good for the production of bulk chemicals.

Because *o*-xylene contains two electron-donating groups, it is highly susceptible to dinitrification side reactions, making it difficult to increase the yield of its mononitrification products beyond 90%. However, mononitro-*o*-xylenes are bulk chemical raw materials with an annual global demand of more than 100,000 tons. Increasing the yield by just a few percentage points can also significantly reduce production costs. Herein, we demonstrated a nitration process of *o*-xylene with high yield and high throughput by using a commercially available continuous-flow reactor. The main impurities of the process were researched in detail. Additionally, the method was also applied to the nitrification of *p*-xylene, toluene, and chlorobenzene.

## 2. Results and Discussion

### 2.1. Optimization of Nitration in Continuous-Flow

The initial experiment was performed to investigate the parameters affecting the reaction greatly, such as temperature, ratio of H_2_SO_4_ to HNO_3_, H_2_SO_4_ concentration, and flow rate. As shown in Figure 2, H_2_SO_4_ was diluted into a certain concentration and mixed with HNO_3_ to prepare the mixed acid. The mixed acid and *o*-xylene were then pumped into the continuous reactor (Corning Advanced-Flow Reactor G1 with five standard fluidic modules). The reaction solution was collected in a vessel. The organic phase was separated and washed with water and brine successively, and then the samples were analyzed by gas chromatography.

The influence of temperature from 50 °C to 110 ℃ was initially studied with the other parameters fixed as follows: H_2_SO_4_ concentration = 70%, H_2_SO_4_/HNO_3_ mole ratio = 1.0, HNO_3_/o-xylene mole ratio = 1.2, and flow rate of *o*-xylene = 10 g/min. With increased temperature, the conversion of *o*-xylene increased. The selectivity of the products peaked at 100 ℃ (Figure 3a). The H_2_SO_4_/HNO_3_ mole ratio was investigated next. Results show that H_2_SO_4_/HNO_3_ = 4.0 gave the best conversion of substrate, and H_2_SO_4_/HNO_3_ = 3.0 gave the best product selectivity (Figure 3b). H_2_SO_4_/HNO_3_ = 3.0 was selected for further study considering that relatively less acid was more favorable for manufacturing. The H_2_SO_4_ concentration was subsequently examined. With increased H_2_SO_4_ concentration, the conversion obviously increased. However, the selectivity of products sharply decreased when H_2_SO_4_ concentration exceeded 70% (Figure 3c). Considering that the nitration is a two-phase reaction, flow rate greatly affected the mass transfer and hence the reaction results [35]. With increased flow rate, conversion and selectivity increased. When the flow rate of *o*-xylene exceeded 10 g/min, the reaction conversion and selectivity were maintained (Figure 3d).

Under the existing conditions, the conversion remained too low. Accordingly, five more reaction modules were added to this system to prolong the residence time (Figure 4). Residence time (*t*) was determined as follows Equation where *V*, *Q_aq_*, and *Q_org_* were the microchannel volume, aqueous volumetric flow rate, and organic volumetric flow rate:

(1) $$t=\frac{V}{Q_{org}+Q_{aq}}$$

Samples were collected at different outlets, which showed that prolonging the residence time improved the conversion to some certain extent. However, the reaction was nearly balanced because conversion from outlet III was very close to that from outlet IV (Table 1).

To break the balance and achieve high product yield, another flow of mixed acid with different concentrations of H_2_SO_4_ and HNO_3_/*o*-xylene was pumped into the system at the sixth module (Figure 5). The addition of mixed acid to the continuous-flow system significantly promoted the reaction (Table 2). To our delight, when the initial mixed acid from pump B and supplemental mixed acid from pump C were 70% H_2_SO_4_ and HNO_3_/*o*-xylene = 1.2, the product yield reached 94.1%, with a throughput of 800 g/h of products (Table 2, entry 3). A comparison experiment with an initial double flow rate of mixed acid was conducted, too. The results show that the direct pumping in double flow rate of mixed acid produced more dinitrification products, resulting in a significant decrease in yield and product selectivity (Table 2, entry 4). 

### 2.2. Research on Impurities

The impurities of this process were also studied in detail. The main impurities were isolated, and their structures were identified by ^1^H NMR and/or X-ray crystal-structure determination. Results indicate that they were all dinitroproducts of *o*-xylene (Figure 6, **7–10**). Unlike this continuous process, another phenolic impurity **11** in the batch reaction process amounted to 2%, which seriously affected the product quality (Figure 5, **11**). The phenolic impurity **11** may have been generated from the oxidation of impurity **7** by HNO_3_. To remove it, a large amount of NaOH aqueous solution was necessary for postprocessing, which produced substantial liquid waste. Interestingly, impurity **11** was diminished to about 0.1% in this continuous-flow process, which enabled the omission of the alkaline solution washing step and reduced the wastewater emission. The sharp decrease in impurity **11** may be correlated with the short reaction time in our continuous-flow process.

### 2.3. Extension to Analogues

To test the generality of the method, the reaction conditions were subsequently extended to the synthesis of other analogues. *p*-Xylene was converted to 2-nitro-*p*-xylene with 93.8% yield under these conditions (Table 3, entry 2). Toluene was converted to nitrotoluenes with 96.0% yield, and its main products were *o*- and *p*- nitro products. Similar to *o*-xylene (Table 3, entry 1), toluene exhibits poor regioselectivity under these conditions (Table 3, entry 3). However, the situation changed a lot in chlorobenzene’s nitration. Chlorobenzene not only obtained a 97.2% mononitration yield, but also obtained >99% para-selectivity (Table 3, entry 4). The slight electron-withdrawing group on the benzene ring rather than the electron-donating group might be the reason for the extremely high regioselectivity.

## 3. Materials and Methods

### 3.1. General Information

All the chemicals were purchased from standard commercial vendors and were used without any further purification. Flash column chromatography was performed using silica gel (300–400 mesh). ^1^H NMR chemical shifts were reported in ppm (δ) relative to tetramethylsilane (TMS) with the solvent resonance employed as the internal standard (CDCl_3_, 7.26 ppm, DMSO-*d_6_,* 2.50 ppm). Gas chromatography was recorded on a Shimadzu GC 2014. Crystallographic data were collected on an Agilent Technologies Germini by using the Cu-Kα radiation (λ = 1.5406 Å) at room temperature. All products and impurities were isolated by flash chromatography on silica gel and characterized by ^1^H NMR. Impurities **7**, **8**, **10,** and **11** were crystallized in ethanol and confirmed by X-ray crystal structure determination.

### 3.2. Batch Nitration Procedure A

Into a 250 mL round-bottom flask equipped with a mechanical stirrer was added 53 g (500 mmol) of *o*-xylene and then cooled to 15–20 °C. 63 g (660 mmol, 1.3 equiv.) Concentrate H_2_SO_4_ (98%) was mixed with 7 mL of ice water and 37 g (600 mmol, 1.2 equiv.) of FNA to make the mixed acid. The mixed acid was then added dropwise into the flask in an hour. The reaction was kept at 15–20 °C for additional 30 min. The organic phase was separated and then washed two times with 50 mL water. It was further washed with 50 mL brine to remove residual water. The products were analyzed using gas chromatography with an HP5 capillary column and a flame ionization detector (FID).

### 3.3. Continuous Flow Reactor Setup 

All flow chemistry experiments were conducted using a Corning Advanced-Flow Reactor G1 setup, consisting of plunger pumps (Corning dual pump KP-22-33DC, Hanbang technology NP7010C), heat exchanger, and fluidic modules. Each fluidic module consists of four confined structured hybrid glass layers that yield three zones for the flow: a reaction zone (of 8.3 mL volume) sandwiched by two heat transfer zones (with an area per unit volume of 788.5 m^−1^). The internal diameter of the reaction channel is 1 mm at its narrowest point and 1 cm at its widest point. Connections between plunger pumps and the microreactor consisted of PTFE tubing (1/4 in o.d.). The recommended flow rates for this device range from 10 to 200 mL/min.

### 3.4. General Procedure B for Condition Optimization of Continuous Nitration

The concentrate H_2_SO_4_ (98%) was mixed with a known quantity of ice-cold water to make the required concentration of H_2_SO_4_. The diluted H_2_SO_4_ was then mixed with a known quantity of FNA to make the mixed acid with desired mole ratio. The two reactants (*o*-xylene and mixed acid) were pumped into the continuous reactor through plunger pumps to react under desired flow rate and temperature. Samples were collected at the outlet in a fixed quantity of ice-cold water. A known quantity of CH_2_Cl_2_ was used to extract the organic phase from these samples. The extracted organic phase was washed two times with water and separated by gravity. It was further washed with brine to remove residual water. The samples were analyzed using gas chromatography with an HP5 capillary column and a flame ionization detector (FID).

### 3.5. General Procedure C for the Long Run of Continous Niration of o-xylene and Its Analogues

The concentrate H_2_SO_4_ (98%) was mixed with ice-cold water to make the 70% H_2_SO_4_. The diluted H_2_SO_4_ was then mixed with FNA to make the mixed acid (10.4 mol/L H_2_SO_4_, 3.5 mol/L HNO_3_). The organic phase was pumped in with a flow rate of 11.4 mL/min from pump A through the first fluidic module. The mixed acid was pumped in from pump B and pump C through the first module and the sixth module with a flow rate of 32.2 mL/min, respectively. The reaction temperature was set to 100 °C and the residence time was 90 s. Samples were collected at the outlet in a fixed quantity of ice-cold water. A known quantity of CH_2_Cl_2_ was used to extract the organic phase from these samples. The extracted organic phase was washed two times with water and separated by gravity. It was further washed with brine to remove residual water. The samples were analyzed using gas chromatography with an HP5 capillary column and a flame ionization detector (FID).

### 3.6. Preparation Procedure D for Impurities 

Into a 25 mL round-bottom flask equipped with a mechanical stirrer was added 5.3 g (50.0 mmol) of *o*-xylene and then cooled to 15–20 °C. A total of 6.3 g (65.6 mmol, 1.3 equiv.) of concentrate H_2_SO_4_ (98%) was mixed with 3.7 g (60.0 mol, 1.2 equiv.) of FNA (98%) to make the mixed acid. The mixed acid was then added dropwise into the flask in an hour. The reaction was kept at 15–20 °C for an additional 30 min. The organic phase was separated and then washed two times with 50 mL water. It was further washed with 50 mL brine to remove residual water. The residue was purified by flash chromatography on silica gel to give the corresponding impurities.

## 4. Conclusions

A continuous-flow nitration process of *o*-xylene and its main impurities was studied in detail. Under the optimal conditions of this continuous-flow nitration process, the yield of products reached 94.1%, with a throughput of 800 g/h. Compared with the batch process, phenolic impurity decreased from 2% to 0.1%, which simplified the postreaction treatment and reduced the wastewater emission. The method was also applied to the nitrification of *p*-xylene, toluene, and chlorobenzene. The good yield, high throughput, and good control of impurities of the proposed process indicated its important industrial application potential.

## Figures and Tables

**Table 1:** Influence of residence time on the reaction ^a^.

Entry	Outlet	Residence Time (s)	Conversion (%) ^b^	Selectivity of Products (%) ^c^	Mol Ratio of 4-Nitro/3-Nitro Product
1	I	33	51.8	91.9	0.83
2	II	55	66.5	94.3	0.83
3	III	77	74.5	94.2	0.85
4	IV	110	76.1	94.1	0.87

^a ^Reaction conditions: T = 100 °C, H_2_SO_4_ concentration = 70%, H_2_SO_4_/HNO_3_ mole ratio = 3.0, HNO_3_/*o*-xylene mole ratio = 1.2, and flow rate of *o*-xylene = 10 g/min; ^b^ conversion was calculated by GC; ^c^ selectivity of products = total yield of 4-nitro and 3-nitro products/conversion.

**Table 2:** The influence of the added mixed acid on the reaction ^a^.

Entry	H_2_SO_4_ Concentration from Pump C (%)	Mol Ratio of HNO_3_/*o*-Xylene from Pump C	Yield (%) ^b^	Selectivity of Products (%) ^c^	Mol Ratio of 4-Nitro/3-Nitro Product
1	70%	0.8	93.2	95.5	0.84
2	70%	1.0	91.3	95.3	0.84
3	70%	1.2	94.1	95.5	0.84
4 ^d^	/	/	85.7	86.0	0.86
5	80%	0.8	88.2	88.3	0.89
6	80%	1.0	91.2	91.5	0.86
7	80%	1.2	83.2	83.2	0.94

^a^ Uniform reaction conditions unless otherwise noted: T = 100 °C, H_2_SO_4_ concentration = 70%, H_2_SO_4_/HNO_3_ mole ratio = 3.0, HNO_3_/*o*-xylene mole ratio = 1.2 from pump B, and flow rate of *o*-xylene = 10 g/min; ^b^ yield was calculated by GC; ^c^ selectivity of products = total yield of 4-nitro and 3-nitro products/conversion. ^d^ reaction conditions: T = 100 ℃, H_2_SO_4_ concentration = 70%, H_2_SO_4_/HNO_3_ mole ratio = 3.0, HNO_3_/*o*-xylene mole ratio = 2.4 from pump B, no mixed acid from pump C, and flow rate of *o*-xylene = 10 g/min.

**Table 3:**
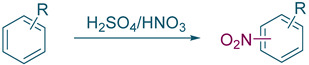
Synthesis of analogues ^a^.

Entry	Substrate	Nitro-Product(s)	Selectivity of Products (%) ^b^	Yield (%) ^c^
1	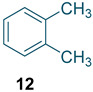	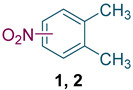	95.5	94.1 (3-nitro = 54.3) ^d^ (4-nitro = 45.7) ^d^
2	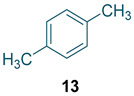	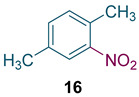	94.0	93.8
3	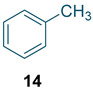	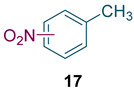	97.3	96.0 (*o* = 59.2) ^d^ (*p* = 40.8) ^d^
4	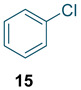	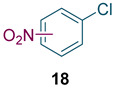	98.9	97.2 (*o* < 1) ^d^ (*p* > 99) ^d^

^a^ Reaction conditions: T = 100 ℃, H_2_SO_4_ concentration = 70%, H_2_SO_4_/HNO_3_ mole ratio = 3.0, HNO_3_/*o*-xylene mole ratio = 1.2 from both pump B and pump C, and flow rate of substrates = 10 g/min; ^b^ selectivity of products = total yield of mononitro-products/conversion. ^c^ yield and selectivity was calculated by GC; ^d^ number in parentheses is regioselectivity.

## Data Availability

The data presented in this study are available in the supplementary material.

## Supplementary Materials

The following supporting information can be downloaded at: https://www.mdpi.com/article/10.3390/molecules27165139/s1, Image of continuous flow reactor setup; ^1^H NMR data and spectrum, character for compound **1**, **2**, **7**–**11**, **16**–**18**; XRD single crystal data for compound **7**, **8**, **10** and **11**.

## Sample Availability

Some of the compounds described in this article are available from the authors.
