# Supplementary material for: Continuous-Flow Synthesis of Nitro-o-xylenes: Process Optimization, Impurity Study and Extension to Analogues
Source: Molecules. 2022 Aug 12;27(16):5139. doi: 10.3390/molecules27165139 (PMC9416712; doi:10.3390/molecules27165139)

Continuous-flow Synthesis of Nitro-o-xylenes: Process Optimization, Impurity Study and Extension to Analogues

Qiao Song [1, 2, *], Xiangui Lei [1, 2], Sheng Yang [1, 2], Sheng Wang [1], Jianhui Wang [3], Jiujun Chen [3], Yong Xiang [3], Qingwu Huang [3], Zhouyu Wang [1, 3, *]

[1] *Department of Chemistry, Xihua University, Chengdu 610039, China*
[2] *Asymmetric Synthesis and Chiral technology Key Laboratory of Sichuan Province, Yibin 644000, China*
[3] *Yinguang Group Sichuan North Hongguang Special Chemical Co., Ltd., Yibin 644000, China*
* Correspondence: songqiao@mail.xhu.edu.cn (Qiao Song), zhouyuwang77@163.com (Zhouyu Wang)

Supporting Information

Contants:

1. Image of continuous flow reactor setup 2
2. Characterization 2
3. Crystal data for impurity 7 and X-ray structure 5
4. Crystal data for impurity 8 and X-ray structure 6
5. Crystal data for impurity 10 and X-ray structure 7
6. Crystal data for impurity 11 and X-ray structure 8
7. ^{1}H spectra 9

1. Image of continuous flow reactor setup

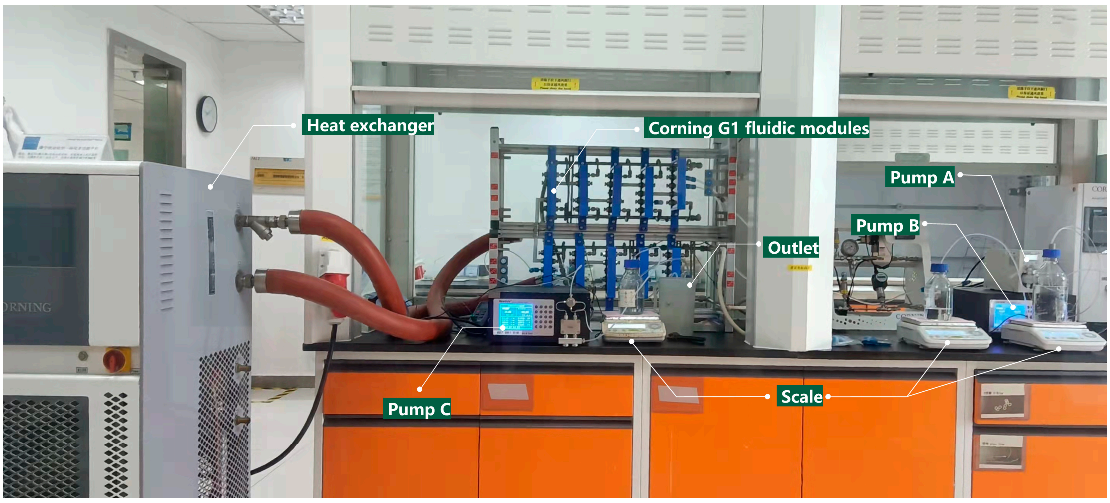

2. Characterization

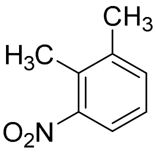

1, 2-dimethyl-3-nitrobenzene (**1**): Prepared according to general procedure C; 43% yield; Yellow oil; **^{1}H NMR (400 MHz, CDCl$_3$)** δ 7.58 (d, *J* = 8.0 Hz, 1H), 7.35 (d, *J* = 7.5 Hz, 1H), 7.19 (t, *J* = 7.9 Hz, 1H), 2.36 (d, *J* = 2.1 Hz, 6H).

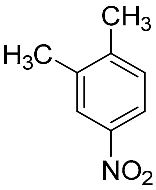

1, 2-dimethyl-4-nitrobenzene (**2**): Prepared according to general procedure C; 51% yield; Yellow solid; Melting point: 29-31℃; **^{1}H NMR (400 MHz, CDCl$_3$)** δ 8.00 (d, *J* = 2.2 Hz, 1H), 7.97 – 7.91 (m, 1H), 7.27 (d, *J* = 8.3 Hz, 1H), 2.67 – 2.18 (m, 6H).

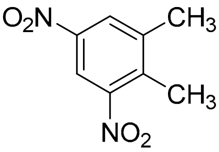

1, 2-dimethyl-3, 5-dinitrobenzene (**7**): Prepared according to preparation procedure D; 11% yield; Yellow solid; Melting point: 115-116℃; **^{1}H NMR (400 MHz, DMSO)** δ

8.12 (d, J = 8.5 Hz, 1H), 7.68 (d, J = 8.5 Hz, 1H), 2.44 (s, 3H), 2.21 (s, 3H).

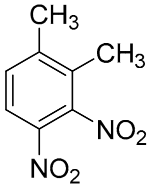

1, 2-dimethyl-3, 5-dinitrobenzene (**8**): Prepared according to preparation procedure D; 18% yield; Yellow solid; Melting point: 89-90℃; **^{1}H NMR (400 MHz, $CDCl_3$)** δ 7.98 (d, J = 8.4 Hz, 1H), 7.44 (d, J = 8.4 Hz, 1H), 2.46 (s, 3H), 2.26 (s, 3H).

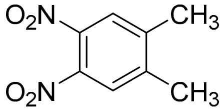

1, 2-dimethyl-4, 5-dinitrobenzene (**9**): Prepared according to preparation procedure D; 19% yield; Yellow solid; Melting point: 74-75℃; **^{1}H NMR (400 MHz, $CDCl_3$)** δ 7.65 (s, 2H), 2.44 (s, 6H).

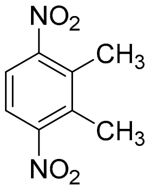

2, 3-dimethyl-1, 4-dinitrobenzene (**10**): Prepared according to preparation procedure D; 22% yield; Yellow solid; Melting point: 80-82℃; **^{1}H NMR (400 MHz, $CDCl_3$)** δ 8.48 (d, J = 2.3 Hz, 1H), 8.24 (d, J = 2.2 Hz, 1H), 2.51 (s, 3H), 2.50 (s, 3H).

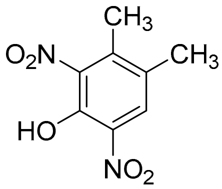

3, 4-dimethyl-2, 6-dinitrophenol (**11**): Prepared according to preparation procedure D; 10% yield; Yellow solid; Melting point: 124-125℃; **^{1}H NMR (400 MHz, $CDCl_3$)** δ 10.66 (s, 1H), 8.02 (s, 1H), 2.34 (s, 3H), 2.29 (s, 3H).

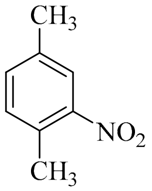

1, 4-dimethyl-2-nitrobenzene (**16**): Prepared according to general procedure C; 93.8% yield; Yellow oil; **^{1}H NMR (400 MHz, $CDCl_3$)** δ 7.79 (s, 1H), 7.47 – 7.25 (m, 1H), 7.23 (d, J = 7.8 Hz, 1H), 2.57 (s, 3H), 2.41 (s, 3H).

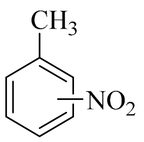

o- and *p*-Nitrotoluene (**17**): Prepared according to general procedure C; 96.0% yield; Yellow oil; **^{1}H NMR (400 MHz, CDCl$_3$)** δ 8.33 – 8.03 (m, 1H), 8.03 – 7.91 (m, 1H), 7.55 – 7.44 (m, 1H), 7.36 – 7.28 (m, 3H), 2.58 (s, 3H), 2.45 (s, 2H).

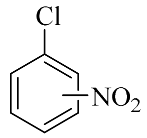

Nitrochlorobenzene (**18**): Prepared according to general procedure C; 97.2% yield; Yellow crystal; Melting point: 83-84°C；**^{1}H NMR (400 MHz, CDCl$_3$)** δ 8.30 – 7.98 (m, 2H), 7.59 – 7.40 (m, 2H).

3. Crystal data for impurity 7 and X-ray structure

Bond precision: C-C = 0.0030 A Wavelength=0.71073

Cell:	a=14.7058(12)	b=3.9648(2)	c=15.7815(13)
	alpha=90	beta=108.504(9)	gamma=90

Temperature: 293 K

	Calculated	Reported
Volume	872.58(12)	872.58(12)
Space group	P 21/n	P 1 21/n 1
Hall group	-P 2yn	-P 2yn
Moiety formula	C8 H8 N2 O4	C8 H8 N2 O4
Sum formula	C8 H8 N2 O4	C8 H8 N2 O4
Mr	196.16	196.16
Dx,g cm-3	1.493	1.493
Z	4	4
Mu (mm-1)	0.122	0.122
F000	408.0	408.0
F000'	408.24	
h,k,lmax	18,4,19	18,4,19
Nref	1768	1763
Tmin,Tmax	0.958,0.970	0.954,1.000
Tmin'	0.958	

Correction method = # Reported T Limits: Tmin = 0.954 Tmax = 1.000

AbsCorr = MULTI-SCAN

Data completeness = 0.997 Theta(max) = 26.367

R(reflections) = 0.0475(1261) wR2(reflections) = 0.1350(1763)

S = 1.032 Npar = 129

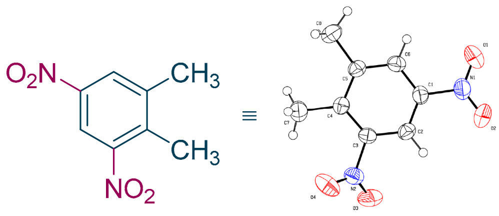

4. Crystal data for impurity 8 and X-ray structure

Bond precision:		C-C = 0.0059 A	Wavelength=0.71073
Cell:	a=8.0068(7)	b=14.4875(14)	c=16.1915(14)
	alpha=90	beta=99.644(8)	gamma=90
Temperature: 293 K			

	Calculated	Reported
Volume	1851.7(3)	1851.6(3)
Space group	P 21/c	P 1 21/c 1
Hall group	-P 2ybc	-P 2ybc
Moiety formula	C8 H8 N2 O4	C8 H8 N2 O4
Sum formula	C8 H8 N2 O4	C8 H8 N2 O4
Mr	196.16	196.16
Dx,g cm-3	1.407	1.407
Z	8	8
Mu (mm-1)	0.115	0.115
F000	816.0	816.0
F000'	816.49	
h,k,lmax	10,18,20	10,18,20
Nref	3772	3768
Tmin,Tmax	0.961,0.972	0.989,1.000
Tmin'	0.961	

Correction method = # Reported T Limits: Tmin = 0.989 Tmax = 1.000

AbsCorr = MULTI-SCAN

Data completeness = 0.999	Theta(max) = 26.369
R(reflections) = 0.0778(2112)	wR2(reflections) = 0.2651(3768)
S = 1.031	Npar = 257

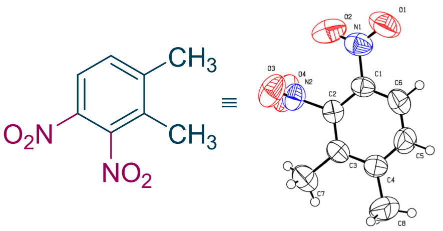

5. Crystal data for impurity 10 and X-ray structure

Bond precision:	C-C = 0.0044 A		Wavelength=0.71073
Cell:	a=9.2591(7)	b=13.2445(12)	c=15.5655(13)
	alpha=107.097(8)	beta=99.113(7)	gamma=95.344(7)

Temperature: 293 K

	Calculated	Reported
Volume	1781.6(3)	1781.6(3)
Space group	P -1	P -1
Hall group	-P 1	-P 1
Moiety formula	C8 H8 N2 O4	4 (C8 H8 N2 O4)
Sum formula	C8 H8 N2 O4	C32 H32 N8 O16
Mr	196.16	784.65
Dx,g cm-3	1.463	1.463
Z	8	2
Mu (mm-1)	0.120	0.120
F000	816.0	816.0
F000'	816.49	
h,k,lmax	11,16,19	11,16,19
Nref	7276	7262
Tmin,Tmax	0.959,0.970	0.564,1.000
Tmin'	0.959	

Correction method = # Reported T Limits: Tmin = 0.564 Tmax = 1.000

AbsCorr = MULTI-SCAN

Data completeness = 0.998	Theta(max) = 26.370
R(reflections) = 0.0684(3324)	wR2(reflections) = 0.2192(7262)
S = 0.999	Npar = 513

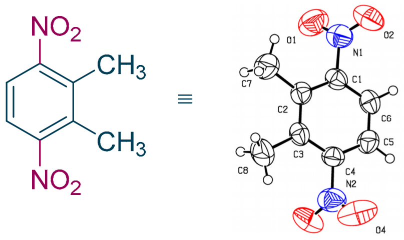

6. Crystal data for impurity 11 and X-ray structure

Bond precision:		C-C = 0.0050 A	Wavelength=0.71073
Cell:	a=10.7951(12)	b=8.1053(9)	c=10.8626(9)
	alpha=90	beta=102.938(6)	gamma=90

Temperature: 293 K

	Calculated	Reported
Volume	934.92(17)	934.92(17)
Space group	P 21/n	P 21/n
Hall group	-P 2yn	-P 2yn
Moiety formula	C8 H8 N2 O5	C8 H8 N2 O5
Sum formula	C8 H8 N2 O5	C8 H8 N2 O5
Mr	212.16	212.16
Dx,g cm-3	1.331	1.331
Z	4	4
Mu (mm-1)	0.128	0.128
F000	440.0	440.0
F000'	440.28	
h,k,lmax	12,9,13	12,9,12
Nref	1708	1700
Tmin,Tmax	0.973,0.986	0.613,0.745
Tmin'	0.970	

Correction method = # Reported T Limits: Tmin = 0.613 Tmax = 0.745

AbsCorr = MULTI-SCAN

Data completeness = 0.995	Theta(max) = 25.330
R(reflections) = 0.0686(954)	wR2(reflections) = 0.1382(1700)
S = 1.034	Npar = 141

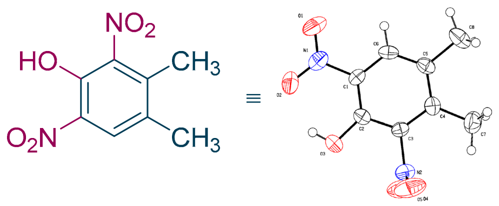

7. ^{1}H spectra

^{1}H NMR spectrum of **1**

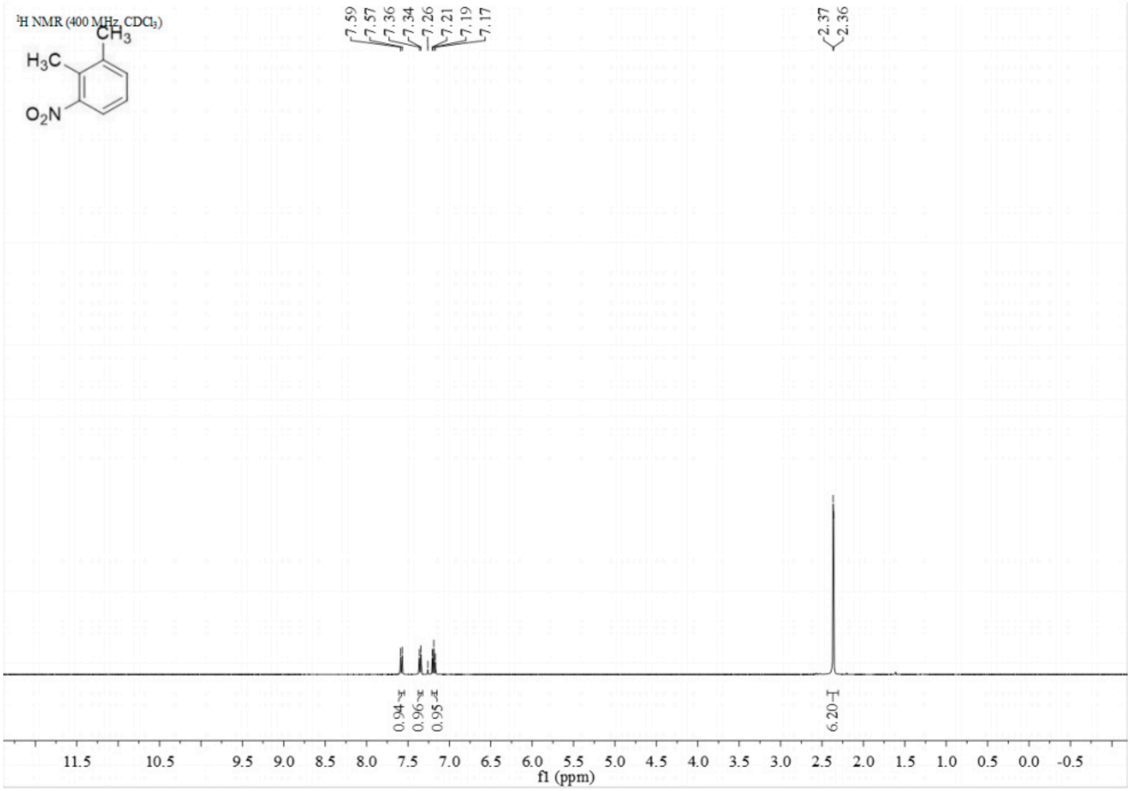

^{1}H NMR spectrum of **2**

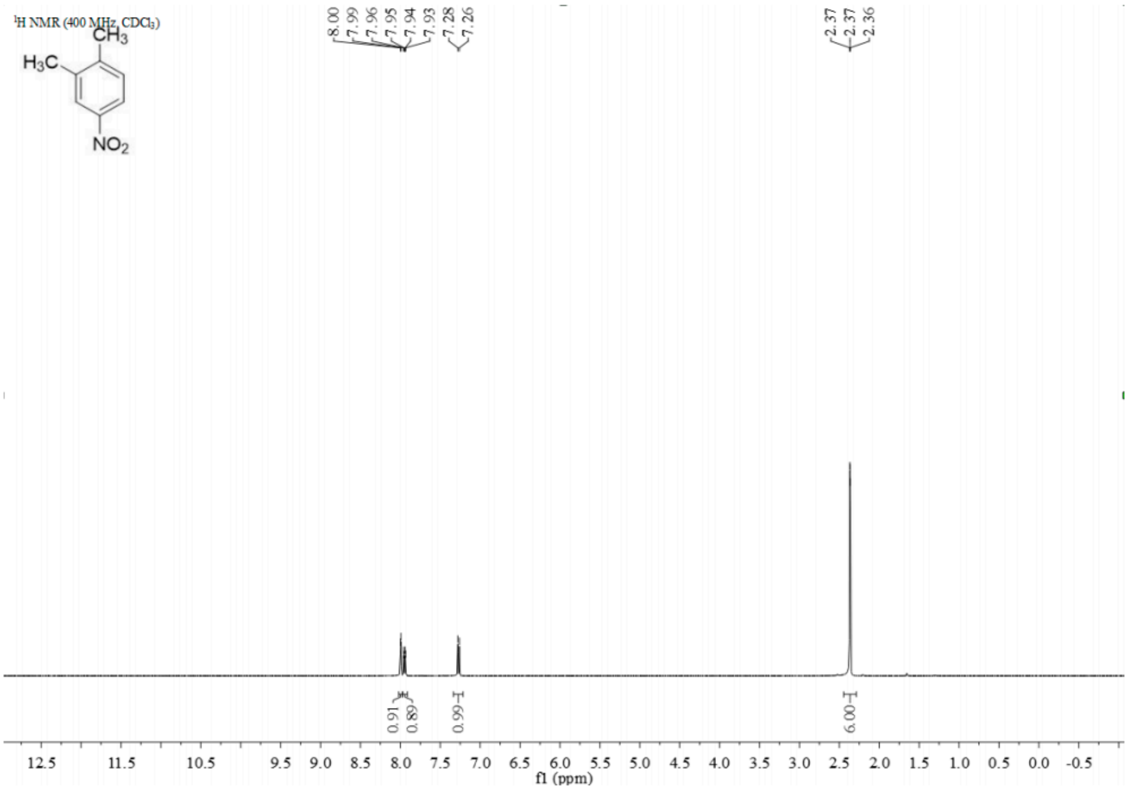

^{1}H NMR spectrum of **7**

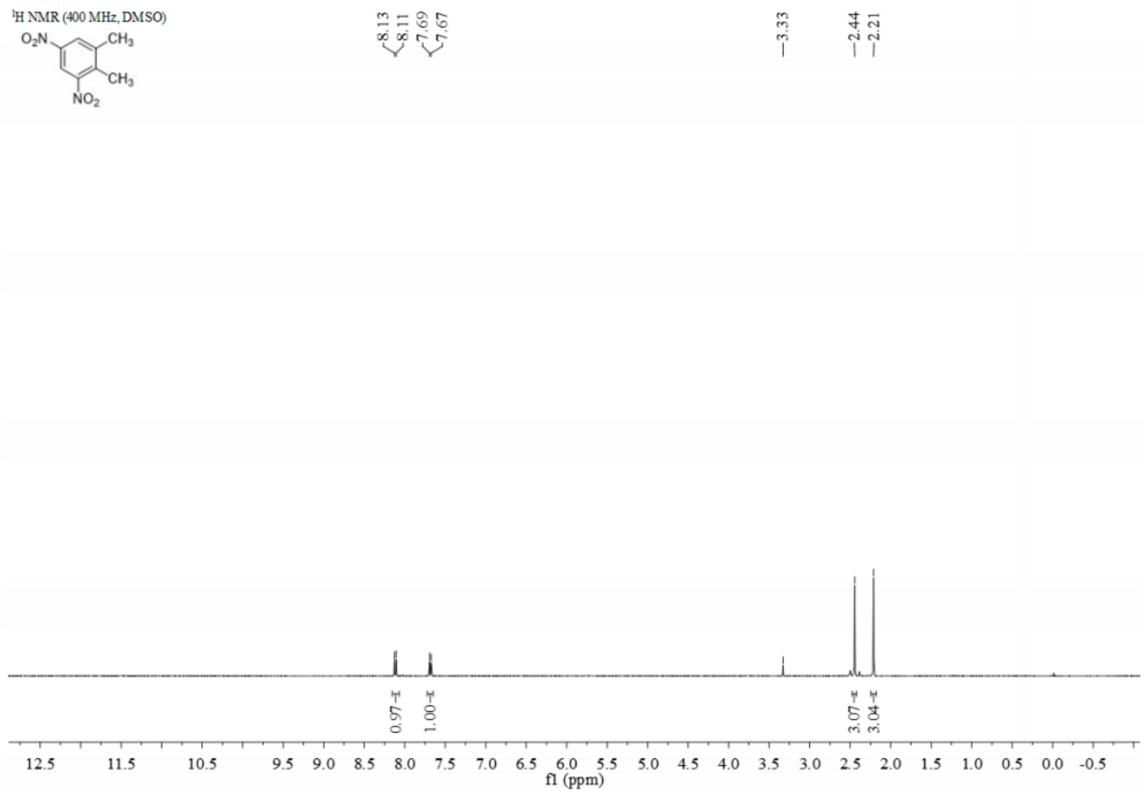

^{1}H NMR spectrum of **8**

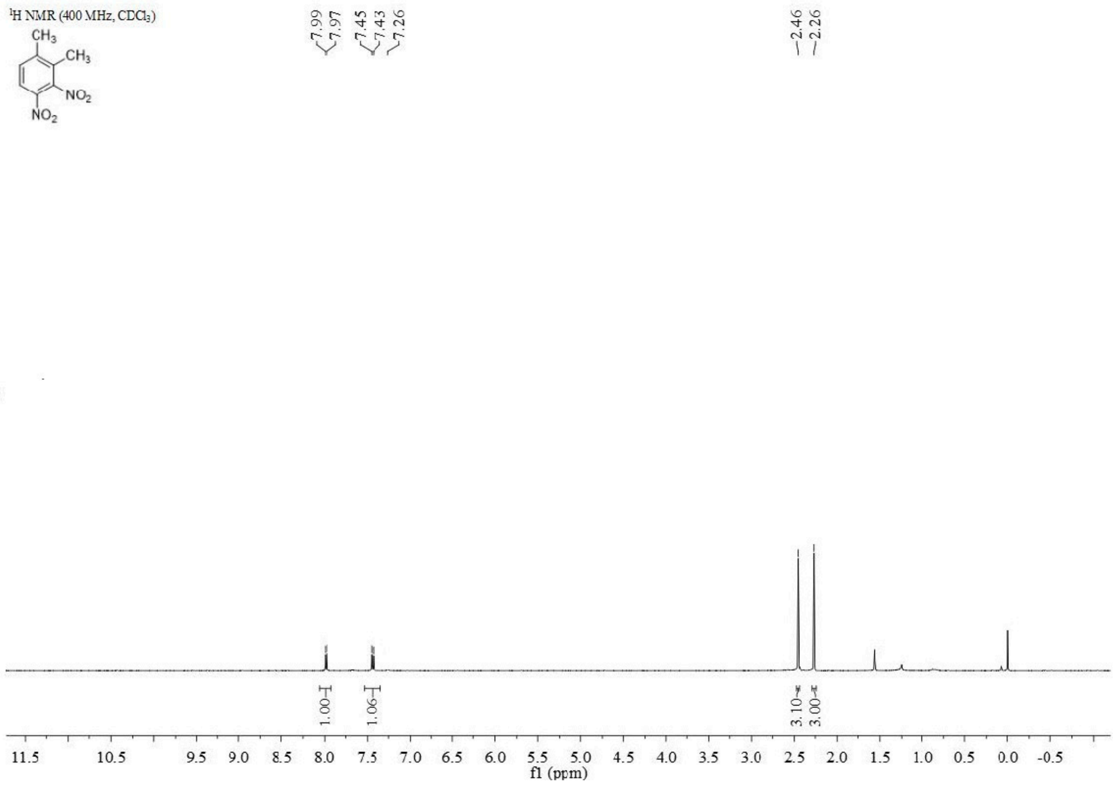

^{1}H NMR spectrum of **9**

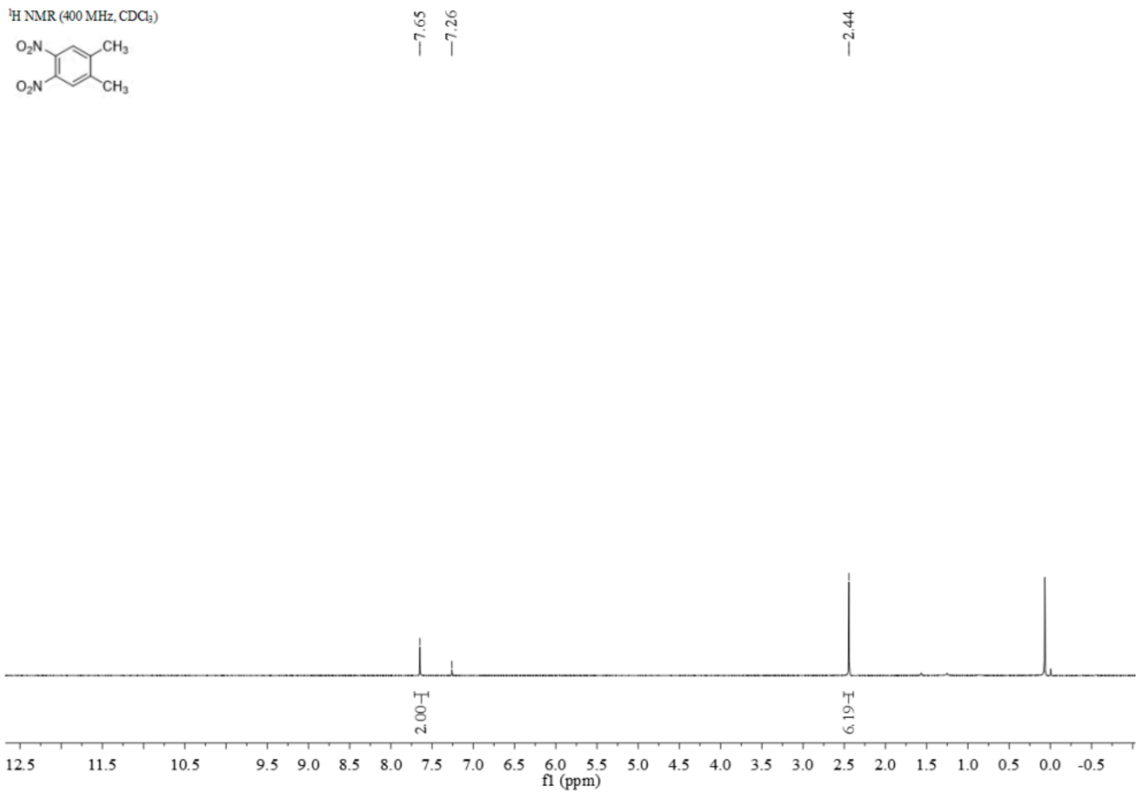

^{1}H NMR spectrum of **10**

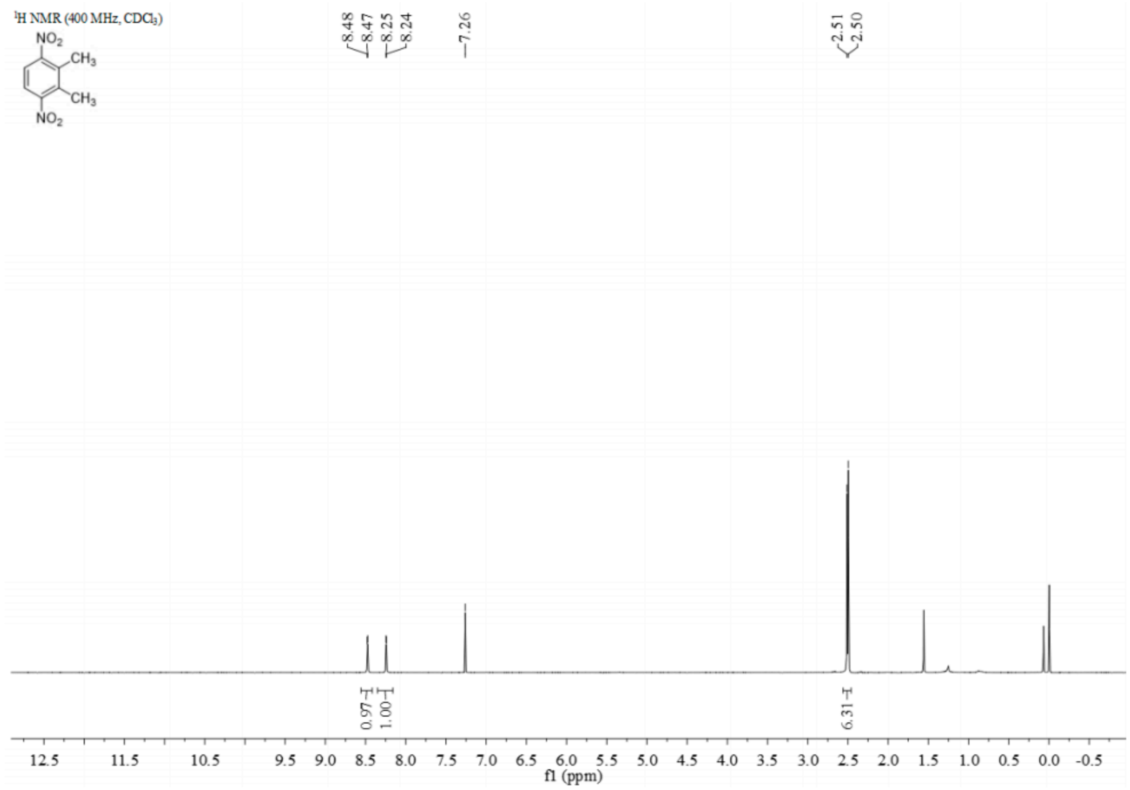

^{1}H NMR spectrum of **11**

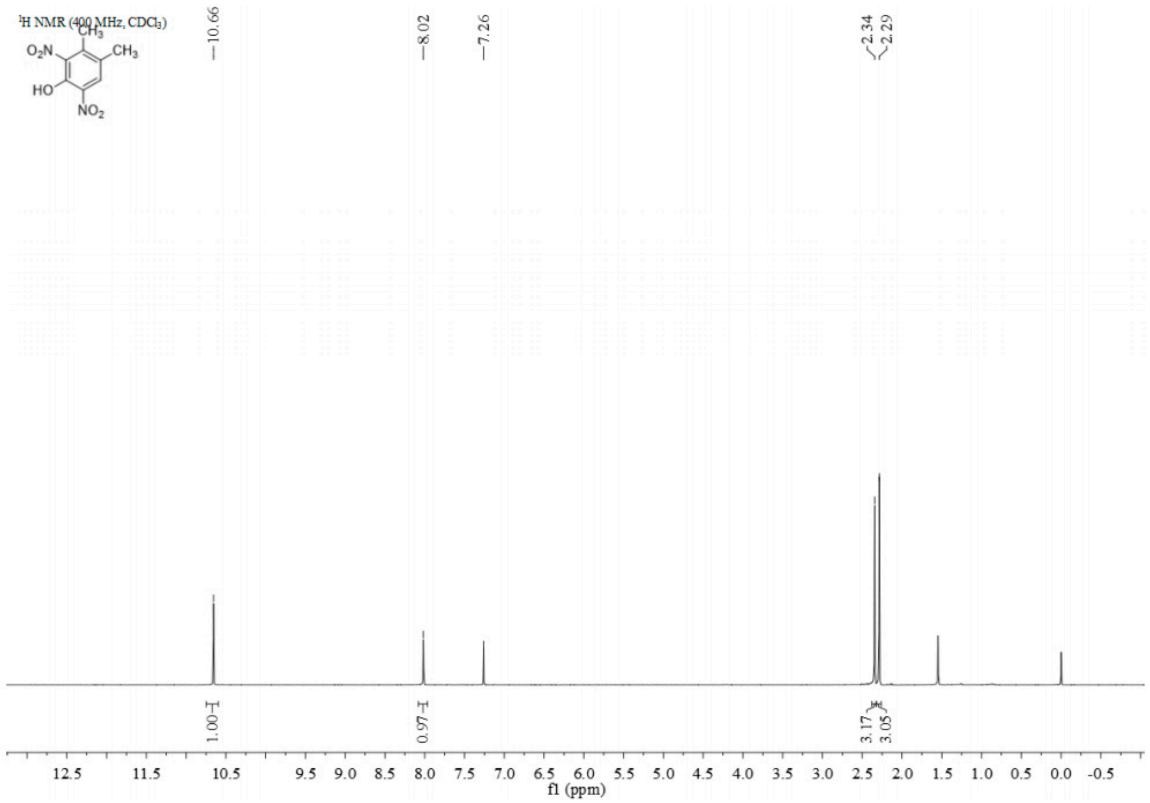

^{1}H NMR spectrum of **16**

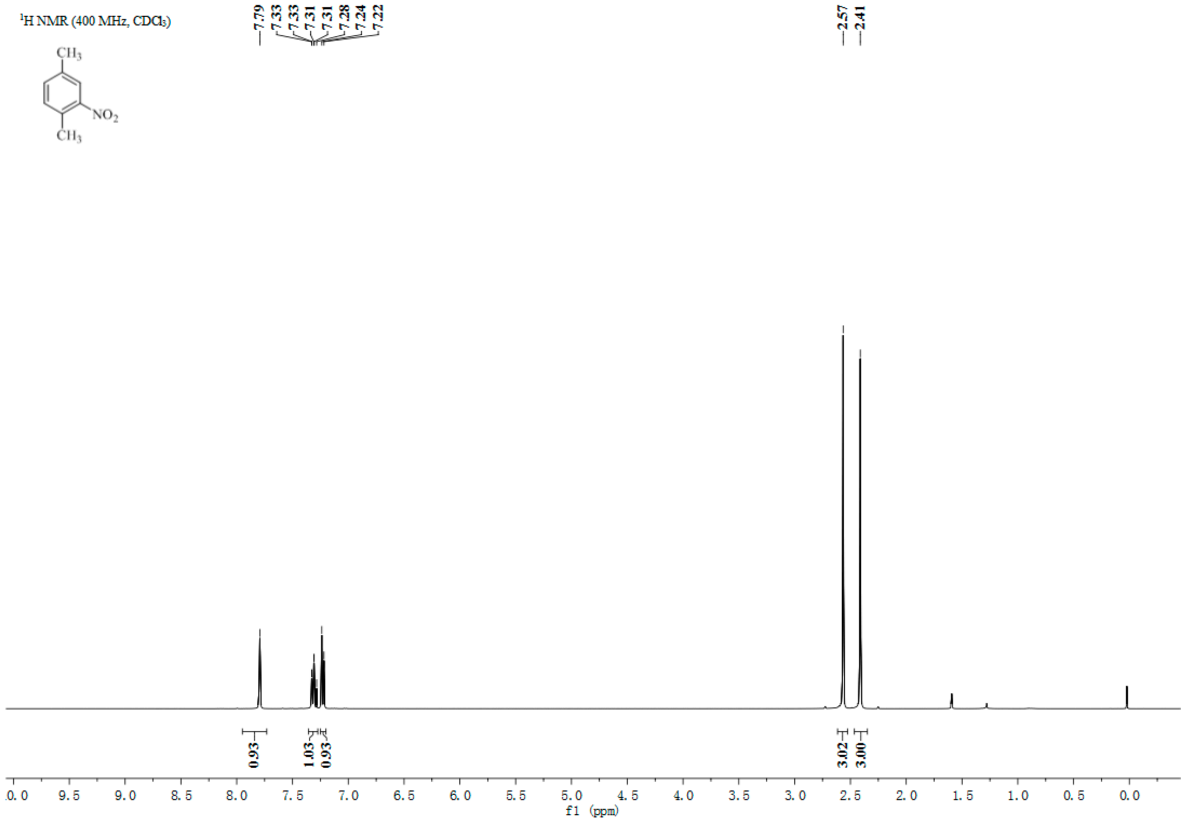

^{1}H NMR spectrum of **17**

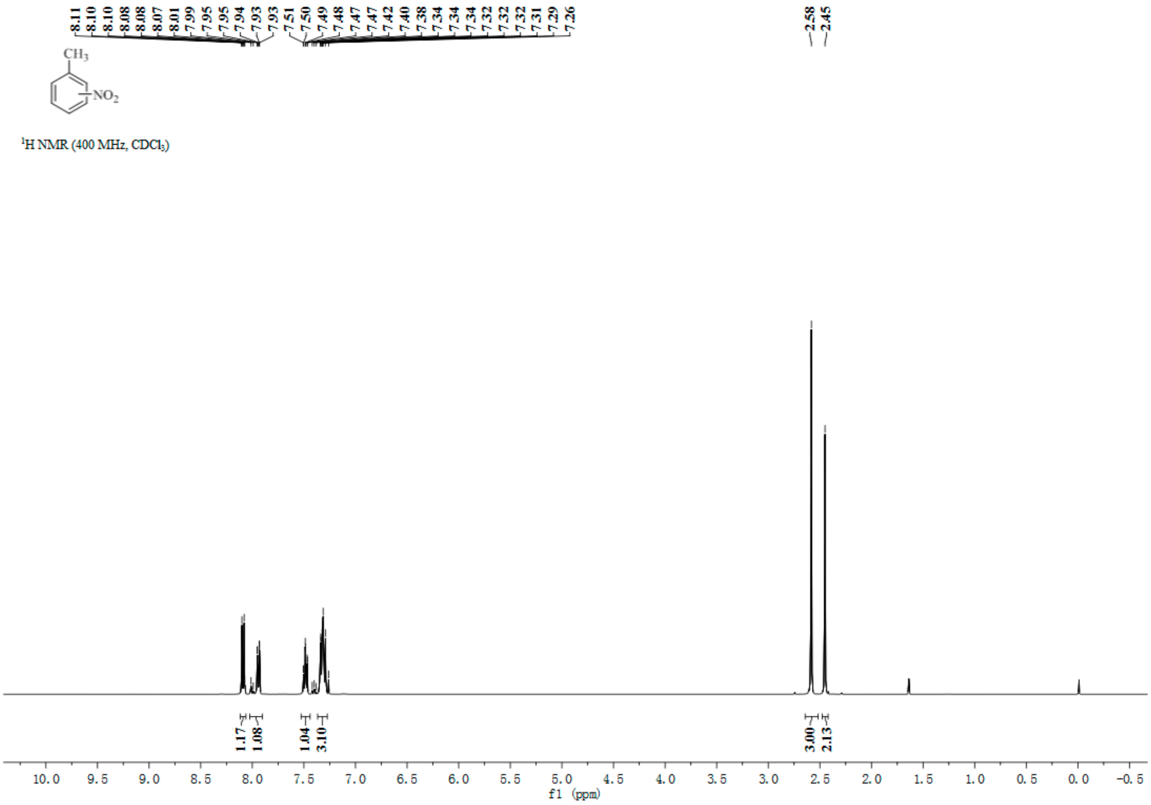

^{1}H NMR spectrum of **18**

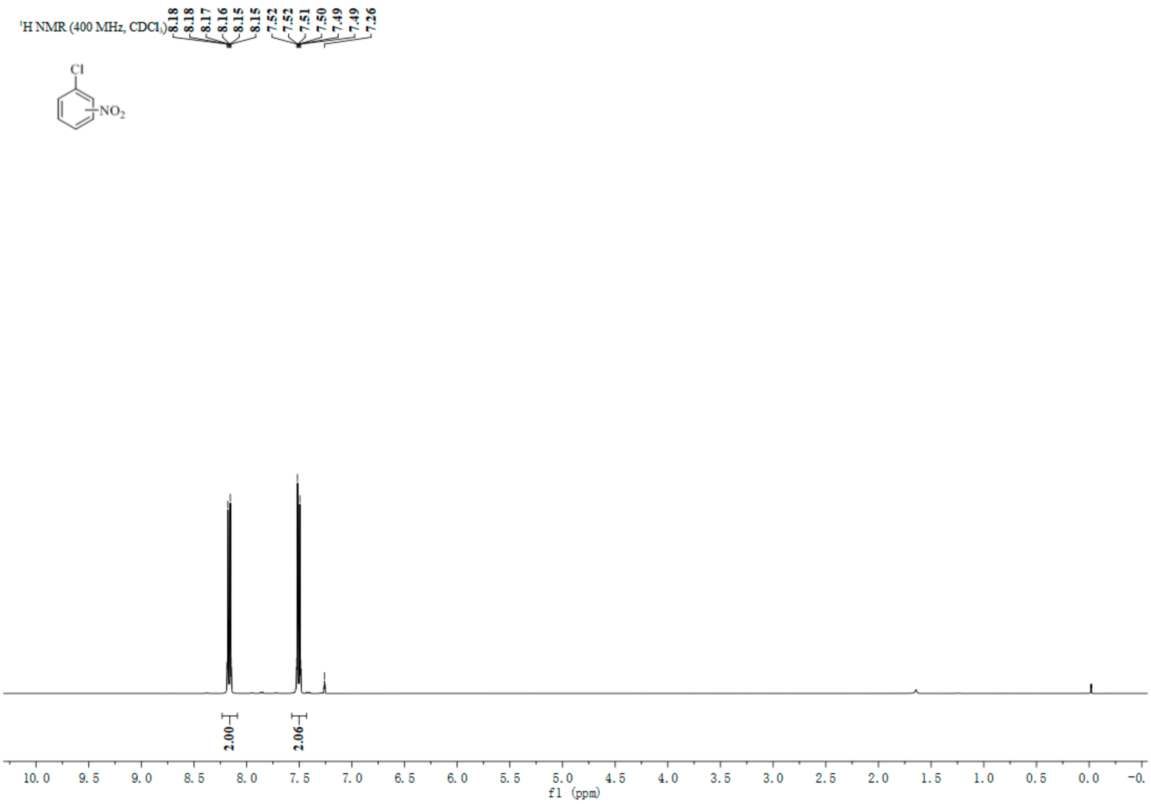

Supplement: Supplementary file 1 [file molecules-27-05139-s001.zip › molecules-1837251-supplementary.pdf]
